# Specific Barriers and Drivers in Different Stages of Decision-Making about Energy Efficiency Upgrades in Private Homes

**DOI:** 10.3389/fpsyg.2016.01362

**Published:** 2016-09-08

**Authors:** Christian A. Klöckner, Alim Nayum

**Affiliations:** Department of Psychology, Norwegian University of Science and TechnologyTrondheim, Norway

**Keywords:** energy efficiency upgrades, private homes, transtheoretical model, decision stages, barriers, drivers

## Abstract

Energy efficiency upgrades of privately owned homes like adding to the insulation layers in the walls, roof or floor, or replacing windows with more efficiently insulated versions can contribute significantly to reducing the energy impact of the building sector and thus also the CO_2_ footprint of a household. However, even in countries like Norway that have a rather high rate of renovation, energy upgrades are not always integrated into such a refurbishment project. This study tests which structural and internal psychological barriers hinder and which drivers foster decision-making to implement such measures, once a renovation project is planned. With a theoretical background in stage-based models of decision-making 24 barriers and drivers were tested for their specific effect in the stages of decision-making. The four stages of decision-making assumed in this study were (1) “not being in a decision mode,” (2) “deciding what to do,” (3) “deciding how to do it,” and (4) “planning implementation.” Based on an online survey of 3787 Norwegian households, it was found that the most important barriers toward deciding to implement energy efficiency upgrades were not owning the dwelling and feeling the right time had not come yet. The most important drivers of starting to decide were higher expected comfort levels, better expected living conditions, and an expected reduction of energy costs. For the transition from deciding what to do to how to do it, not managing to make a decision and feeling the right point in time has not come yet were the strongest barriers, easily accessible information and an expected reduction of energy costs were the most important drivers. The final transition from deciding how to do the upgrades to planning implementation was driven by expecting a payoff within a reasonable time frame and higher expected comfort levels; the most important barriers were time demands for supervising contractors and—again—a feeling that the right point in time has not come yet. Implications for policy-making and marketing are discussed.

## Introduction

Providing shelter, which means construction, maintenance, heating and cooling of buildings, is one of the main contributors to the CO_2_ footprint in most countries of the world (Hertwich and Peters, 2009). In the Norwegian context, where the data of this study was collected, housing and the energy used to sustain a comfortable indoor climate are the second most impactful category of household actions (Steen-Olsen et al., 2016) after transportation. Most of the housing-related energy use in Norway is connected to heating. Typically, the older part of the Norwegian housing stock is characterized by poor insulation in spite of the harsh climate and decentralized electric resistance heaters, an unfortunate situation caused by an abundance of cheap electricity produced by water power at the time the houses were constructed. However, in recent years, electricity prices and electricity demand have increased in Norway which at times leads to import of electricity from nuclear or fossil sources. This development combined with the high demand for valuable Norwegian waterpower in the European electricity market has put the energy efficiency of the old Norwegian building stock on the policy agenda.

The energy efficiency of a dwelling can be increased dramatically by upgrading the insulation standard, thus reducing energy loss to the environment. Possible measures are manifold, and best results are achieved by combining several methods, such as substantially increasing the insulation layer in the outer walls, toward the cold loft and/or the basement, as well as installing windows with a higher energy standard. Whereas the energy standard of new buildings has improved by implementing stricter building regulations that for example demand zero emission standards, a large fraction of the building stock constitutes of older buildings which do not fulfill modern standards (Lee and Yik, 2004). Voluntary investments in the older building stock need to take place if national and international goals of lowering the energy intensity of the building sector should be achieved. In a housing market like the Norwegian, where the majority of dwellings are privately owned (Hauge et al., 2013), the focus shifts to the individual homeowner. Owners regularly invest in maintenance and repair of their dwelling. In Norway, 6.3% of the households are engaged in a refurbishment project every year which includes at least one of the following measures: Changing the façade on at least half of the dwelling's outer walls, changing the tiling or other substantial measures regarding the roof or the loft, changing at least half of the dwelling's window area, or substantial measures regarding the foundation wall or the floor toward the basement or the ground (Klöckner and Nayum, 2015). 1.9% combine at least two such measures. This means that a significant fraction of the privately owned building stock is under rehabilitation at any given time. However, only about half of these projects include energy efficiency upgrades. This gap is important because for investments in insulation to be cost effective in the Norwegian situation with still relatively low energy prices, energy efficiency upgrades need to be integrated into an already scheduled refurbishment. Thus, knowledge about the decision-making process is essential to increase the rate by which privately owned dwellings are updated to today's insulation standard.

A substantial number of studies have addressed the challenge of motivating private house-owners to upgrade the energy efficiency of their homes (see Wilson et al., 2013; Friege and Chappin, 2014). Many of these barriers and drivers can be understood as structural, as they refer to the context of decision-making. A limited number of barriers and drivers, however, might also be referred to as internal or psychological, because they refer to inner states, cognitions or mindsets which either foster progression in decision-making or lead to being locked at a certain stage or regressing to earlier stages. Many of the structural barriers and drivers address the cost structure. Higher costs associated with an energy efficiency upgrade and not having the necessary financial resources are identified as a major obstacle (Black et al., 1985; Jakob, 2007; Nair et al., 2010; Rosenow and Eyre, 2013). Jakob (2007) for example studied energy efficiency investments of private homeowners in Switzerland with two samples of 360 and 1046 households and found that the annual tax burden as a proxy of the available income is a good predictor of such investment decisions. Nair et al. (2010) studied 3000 Swedish homeowners and found that income and perceived energy costs were relevant predictors of the decision to invest in energy efficiency measures rather than preferring no-cost measures. While upfront costs are seen as of great importance, beliefs about possible energy savings, therefore a reduction of energy costs and payoff of the investment within a reasonable time frame, and increased market value after energy efficiency investment measures are suggested to be facilitators (Cirman et al., 2011; Zundel and Stieß, 2011; Organ et al., 2013). Zundel and Stieß (2011) surveyed 1008 German households and found that payoff of the investment and retained market value of the house were among the most important drivers, along with increased expected comfort. On the other hand, as two sides of the same coin, concerns about the saving potential for energy costs after an upgrade measure are seen as a barrier to adopting the measure in many studies (Jakob, 2006; Farsi, 2010; Vergragt and Brown, 2012). Closely associated with the financial considerations, policy regulations and monetary instruments like subsidies, low-interest loans, and (taxing) discount are often suggested as boost for potential energy efficiency upgraders since they alleviate the home occupants' financial burden at the time of energy efficiency upgrade projects (Schipper et al., 1985; Shorrock, 2001; Boardman, 2004). However, the effectiveness of such regulations and financial incentives on energy efficiency investment measures is found to be mixed (Nair et al., 2010; Hauge et al., 2013; Friege and Chappin, 2014). It is found that potential upgraders with higher income, who happened to be the owner of the house, prefer major energy efficiency upgrade measures more often than renters, who cannot determine energy efficiency upgrade measures and get the advantages of relevant policy regulations and financial incentives (Black et al., 1985; Banfi et al., 2008).

Also, the perception that information on efficiency measures is credible/trustworthy and easily accessible is suggested to be a driver to the adoption of cost-effective energy efficiency measures; while conflicting, imperfect or biased information plays the opposite role (Wilson et al., 2013). In their literature review, Wilson et al. (2013) furthermore claim that non-monetary factors are systematically understudied in the domain of energy efficiency investments which might bias the findings toward overemphasizing economic motivations. Along that line of argument, lack of access to competent and credible contractors, previous negative experience, and perceived inconvenience of supervising the contractors are categorized as other important non-economic barriers (Vergragt and Brown, 2012; Weiss et al., 2012). Moreover, anticipated conflict with local or national building protection agencies as well as with neighbors due to possible building alterations and extension can hinder energy upgrade measures (Jakob, 2007; Nair et al., 2010). Further, plans about moving home shortly, either due to dissatisfaction with the home or neighborhood or simply due to own capabilities, are found to hinder owners or tenants to carry out specific types of energy efficiency upgrade measures (Matschoss et al., 2013).

Although economic and non-economic structural considerations like the ones introduced in the last two paragraphs are regarded as of prime importance for an energy efficiency upgrade, non-structural aspects have received increased attention (see Wilson et al., 2013; Friege and Chappin, 2014). These types of barriers and drivers are a step toward the internal mechanisms as they refer to expectations of benefits or obstacles which are not clearly placed in the context. A range of co-benefits from energy efficiency upgrades like higher comfort levels, better living conditions, and positive health effects are found to facilitate decisions about energy efficiency upgrade (Jakob, 2006; Farsi, 2010; Zundel and Stieß, 2011; Organ et al., 2013). Meanwhile, anticipated hassles such as disruption to everyday life, stress, and inconvenience resulting from the renovation project are found to impede or delay the decision (Weiss et al., 2012). An interesting purely psychological barrier that came up in the pilot studies (Klöckner et al., 2013; Klöckner, 2014b) is the feeling of not being able to make a decision and tendencies to procrastinate the decision by constantly feeling that the right point in time has not come yet. The feeling of not being at the right point in time to make such a decision or the difficulty of coming to a conclusion are conceptually different from the other barriers explored as primarily are internal, rather than external barriers. They characterize a mindset of continued contemplation or even procrastination, which may be an efficient hinder of progression to more concrete stages of planning. Zundel and Stieß (2011) identified a comparable type of barrier namely that the household member indicated that up to now, he or she did not take the time to deal with this decision. To our knowledge, these barriers have not been studied much in the context of energy efficiency upgrades before, although they might pose a powerful psychological mechanism to end the decision process before it comes to a conclusion. The driver “perceiving the current energy standard as a waste of energy,” which also emerged in the pilot studies, is an internal driver which holds a strong normative component of frugality (see Fujii, 2006, for a discussion of frugality in the environmental domain). If people perceive using more energy than necessary as morally wrong, and they see that they are doing that because the building lacks insulation, this feeling might drive the decision to engage in an energy efficiency upgrade.

The decision about energy efficiency projects such as upgrading the insulation standard “is shaped by an alliance of economic and non-economic motives and goals” (Zundel and Stieß, 2011, p. 91). However, how the various barriers and facilitators influence the decision-making process of potential insulation standard upgraders remains unclear. Whereas many psychological studies about pro-environmental behavior and its drivers utilize behavior models like the theory of planned behavior (Ajzen, 1991), the norm-activation theory (Schwartz and Howard, 1981), the value-belief-norm-theory (Stern, 2000), or a derivate of them (Bamberg and Möser, 2007; Klöckner, 2013), a shift toward a more dynamic understanding of decision-making can be noted in recent papers. Drawing on health psychological stage models like the transtheoretical model (Prochaska et al., 1992; Prochaska and DiClemente, 1994), Bamberg proposed a stage-based model of self-regulated behavior change (Bamberg, 2007, 2012, 2013a,b). The core assumption of this model is that behavior change is not a one-step process but is going through distinct stages, each one answering a specific question about the change process. In the first stage (named *predecision stage* in Bamberg's model), the question to answer is “why do I need to act.” In the second stage (named *preaction stage*), the question is “what can I do.” In the third stage (named *action stage*) the question to answer is “how do I implement this decision.” In the final stage (named *postaction stage*) the question is how to recover from relapses and how to stabilize the behavior. A similar line of thinking has been applied to investment decisions such as buying an electric vehicle (Klöckner, 2014a). In that case, the stages needed to be adapted to the non-repetitive nature of the behavior: The first stage, which corresponds to the predecision stage in Bamberg's model, is characterized by not being interested in acting, which can be interpreted as not being in a cognitive mode of considering a decision (in case of Klöckner, 2014a, buying an electric vehicle or a vehicle at all). The second stage (pre-actional) is characterized by finding out which electric car to buy after having made a general decision for an electric car, whereas the third stage (actional) was characterized by the detailed planning of the ordering process, which ended in the last stage by the purchase. With the adapted stages, the pre-decision dynamics of the investment in the different stages were clearly demonstrated in the longitudinal study by Klöckner (2014a).

For the investment decision under study in this paper, the stages again need to be adjusted to the type of decision. We decided to name the first stage “*not being in decision mode*” which corresponds with respect to the mindset to the pre-decision stage. People do not even think about the behavior at hand. The second stage was named “*deciding what to do*,” which has a clear link to the pre-actional stage: people consider alternatives and explore the options they have. The third stage was named “*deciding how to do it*,” as now the planning gets more concrete and more detailed decisions about the behavioral alternative prioritized in the previous stage are made. This is a stage that would be positioned between the pre-action and the action stage in Bamberg's model as the planning gets more refined, but the implementation decisions are not made yet. In the final stage “*deciding how to implement*,” such implementation arrangements are made, which corresponds to Bamberg's action stage. In the “*not being in decision mode*” stage, which the majority of homeowners is in at a given point in time, no thoughts are spent on energy efficiency upgrading or rehabilitating the dwelling. However, some people leave this mode at some point in time and start considering an energy upgrade, which places them in the “*deciding what to do*” stage. Here they need to decide between for example upgrading the insulation of the walls or replacing the windows. If they reach a decision, what to do, it needs to be decided in the third stage, how to do it, for example by selecting the type of windows. The last stage is planning how to implement this decision concretely, for example by contacting contractors and making arrangements. Applying such a stage-based approach allows for testing if some barriers and drivers are more relevant in early stages of this decision process, whereas others become relevant fist after some early stage decisions have been made. None of the studies cited above chose an approach differentiating by stages of decision-making, which means little is known about when exactly certain barriers and drivers become relevant in decision-making. Thus, this study tests such stage-based differences in the relevance of barriers and drivers of decision-making.

**Figure 1:**
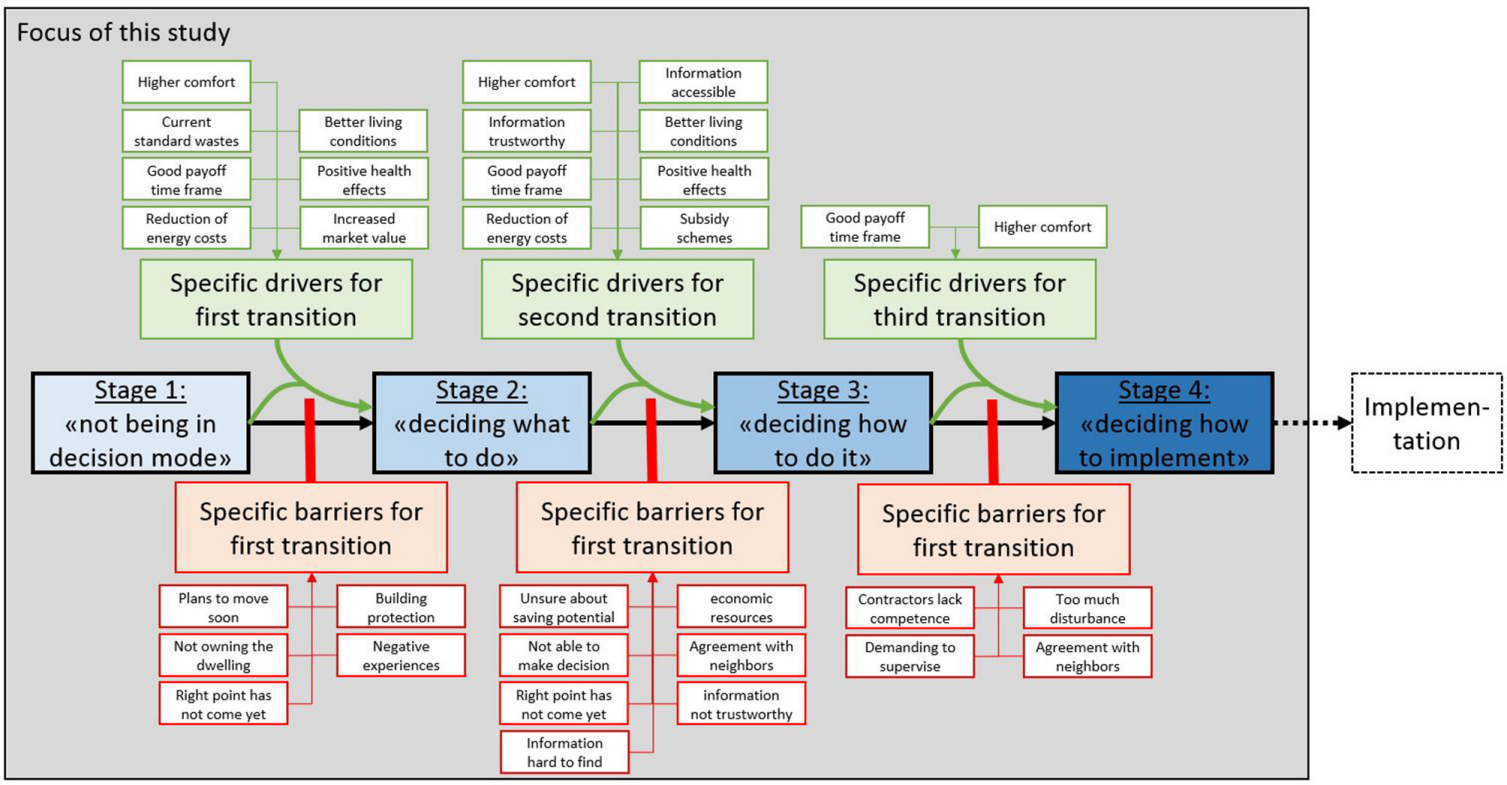
**Theoretically derived structure of stage-specific barriers and drivers**.

Derived from theory, we have assumptions about which barriers and drivers should be most relevant in which stage of the decision-making process. Table 1 displays these assumptions. The list of barriers and drivers was identified based on earlier studies (Klöckner et al., 2013; Klöckner, 2014b) and the literature review presented above. We expected the following barriers to be relevant primarily in the first transition (from *not being in decision mode* to *deciding what to do*) because they pose structural obstacles that prevent most people from starting an energy upgrade project: Planning to move soon, not owning the dwelling, and building protections. Furthermore, we expect that a feeling of not being at the right point yet to start thinking about such a project and having negative experiences from before should also be effective stoppers of a decision-making process at an early stage as they fundamentally undermine the willingness to spend cognitive energy on making this decision. Most drivers should be relevant though at this early stage. We expect only access and trustworthiness of information as well as existing subsidy schemes of being relevant not immediately since they are related to the more concrete planning in later stages. For the transition from *deciding what to do* to *deciding how to do it* many more barriers should become relevant such as insecurity about saving potentials, limited economic resources, feeling of not being the right point in time or lacking the ability to make a decision, but also access to information and doubts about the trustworthiness. Finally, also having to coordinate with neighbors should become relevant for this transition. All of these aspects come into play when planning gets more concrete, and the realization of the chosen alternative is thought through. We expected all but two drivers to be relevant, namely expecting an increased market value and perceiving the current energy standard as a waste of energy. For both, we did not expect any impact in this second transition, because we perceive them as being rather fundamental for starting the process, but then for selecting any alternatives, they seem not to be helpful on the general level. For the last transition from *deciding how to do it* to *deciding how to implement*, we expect lacking competence of contractors, high anticipated effort to supervise them, need for agreement with neighbors and too much anticipated disturbance of everyday life being the relevant barriers, because these are related to very concrete implementation issues of such a project. Two drivers might still be relevant in the implementation stage, but none of them should have a strong impact: Adjustments in expected payoff may happen at this late stage when concrete plans are made and some concrete decisions about measures might give new expectations about comfort improvements. Figure 1 displays the structure of expected stage-specific barriers and drivers. Each barrier is expected to reduce the link between high embracement of one stage description and high embracement of the next stage's description. In other words, they should block the transition at least partly. Each driver is expected to strengthen the link and thus carry the decision maker over the barriers.

**Table 1:** **Hypotheses about the stage-specific relevance of the tested barriers and drivers**.

	**Stage 1->2**	**Stage 2->3**	**Stage 3->4**
**BARRIERS**			
Unsure about the saving potential for energy costs after an upgrade		++	
Plans to move soon	++		
I do not manage to make a decision for what to do		++	
I do not own the dwelling	++		
The right point in time has just not come to upgrade	++	+	
Building protection regulations prevent me from upgrading	++		
Not enough economic resources		++	
Contractors who could do the job lack the necessary competencies			++
Depending on agreement with neighbors		+	+
Difficult to know if information about energy upgrades can be trusted		++	
Too much disturbance of the everyday life through such a project			++
Information about upgrading is difficult to find		++	
Demands much time to supervise the contractors			++
Negative experience from previous projects	++		
**DRIVERS**			
Reduction of energy costs expected after upgrade	++	++	
Increased market value of the dwelling expected after upgrade	++		
Payoff of the investment within a reasonable time frame	+	+	+
Positive health effects expected after upgrade	++	++	
The building standard of the dwelling is perceived as a waste of energy	++		
Better living conditions in the dwelling expected after upgrade	++	+	
Higher comfort levels expected after upgrade	++	++	+
Information about energy upgrade is easily accessible		++	
Information about energy upgrade is trustworthy		++	
There are subsidy schemes in place supporting the upgrade		++	

++ very relevant,

+ relevant.

## Materials and methods

The analyses reported in this paper are part of a larger scale research project on psychological and structural determinants of upgrading the insulation standard of privately owned houses. Other aspects not related to the research question of this paper are reported elsewhere (Klöckner et al., 2013; Klöckner, 2014b; Klöckner and Nayum, 2016). The following sections first describe the sample, the measurement instruments that were used for the analyses, and the analysis strategy.

### Sample

In January-March 2014, TNS Gallup Norway recruited 3787 members of their online panel to answer a questionnaire on refurbishment, energy efficiency upgrades, and their determinants. 2605 of these participants were recruited as a representative household sample, and an additional 1182 were recruited who were either just in the process of considering and planning a refurbishment of their private home or had just undergone such a project. TNS Gallup has ethical clearance for operation of the panel by data protection authorities in Norway and participation was voluntary. TNS Gallup does not disclose any identifying information about the participants to the researchers, so their anonymity in the data material is secured.

For this paper, representativity of the sample was not essential, and priority was rather to have enough participants that were in later stages of decision-making. Thus, the two samples were combined. 49.5% of all respondents were female, 50.5% male, and the mean age was 49.7 years (*SD* 15.5). 51.1% were working full time, 9.5% part-time, 3.1% were self-sustaining, 16.6% pensioners, 9.9% on social support and 7.2% under education. The remaining 2.6% reported another occupational status. 75.4% owned their dwelling, 11.7% owned through a housing cooperation and 12.9% rented their dwelling. 77.4% were living in houses (58.1% single houses, 6.6% twin-house, 12.6% terraced house), 19.5% were living in apartment houses, 3.2% reported other housing types. Both the high fraction of houses and owned dwellings are typical for the Norwegian housing market.

### Measurement instruments

The analyses in this paper use three types of variables from the survey: statements about the state of change, statements about drivers of insulation upgrades and statements about barriers toward insulation upgrades. All three types of variables were included in the online survey.

#### Definition of substantial energy efficiency upgrades

In the survey, it was first defined what the authors understood as a significant energy upgrade of a building. This included at least one of the following measures: (1) Additional insulation of the roof or loft (at least 10 cm additional insulation), (2) Additional insulation of the outer walls (at least 5 cm additional insulation), (3) Changing to extra energy-saving windows (*U*-value 1,0 or lower or triple glazing windows), or (4) Additional insulation of the foundation walls or the floor toward the basement or ground (at least 5 cm additional insulation). This definition was developed in close collaboration with the funding energy efficiency agency based on what they considered an energy upgrade that will deliver energy savings substantial enough. The time frame for all questions in this survey were the next 3 years since refurbishment projects usually are planned and implemented across time spans of more than 1 year.

#### Measure of stage of decision-making

Each stage in the chain of decision-making is—according to the transtheoretical model—characterized by a specific mindset. A statement reflecting each of these mindsets was formulated, and agreement to the statements was measured on a seven-point agreement scale (1 = do not agree at all, 7 = completely agree). The participants had to rate all four statements which was necessary to analyze the structure of the correlations between the statements. Participants were not located in one of the stages for this study, but rather their embracement of the statements characterizing each stage was recorded for all stages. These ratings could either correspond to each other or deviate, and the analysis of these correlation patterns forms the basis for the present study. The following statements were used:

- I do not have plans to change the insulation standard of my dwelling within the next 3 years.” (indicating *not being in decision mode*—reverse coded for the analyses)
- It is decided that something has to be done with the insulation standard, and I will make an effort finding out what can be done within the next 3 years” (indicating *deciding what to do*, hence selecting which measures to implement)
- It is decided that the dwelling's insulation standard needs to be upgraded and how this will be done. Planning of the concrete implementation will be done within the next 3 years.” (indicating *deciding how to do it*, hence selecting concrete methods)
- I have concrete plans for upgrading the insulation standard of the dwelling and the plans will be realized within the next 3 years.” (indicating *deciding how to implement*, hence planning implementation)

The logic behind the analyses presented below is as follows: A successful progression through all stages of the chain would be indicated by high congruency between the four measures (the first one being reversed). A barrier might interfere and reduce the congruency between two successive statements in the chain; a driver will make the relation between successive statements stronger. Based on the statement the participants embraced the most, 79.1% were most likely in the first stage, 8.8% in the second, 4.3% in the third, and 7.8% in the last stage of planning.

#### Measures of drivers and barriers

Ten potential drivers of the decision to upgrade the energy standard of a dwelling were included in the study. Statements for each driver were formulated and presented randomly between other variable indicators not used for the purpose of this study. Agreement to the statements was measured on a seven-point scale (1 = do not agree at all, 7 = completely agree). The drivers were identified in earlier studies (Klöckner et al., 2013; Klöckner, 2014b) and the literature review presented above. Parallel to the drivers, 14 potential barriers were measured in the study. The barriers were also identified in earlier studies (Klöckner et al., 2013; Klöckner, 2014b). The barriers and drivers included are listed in Table 1.

### Analysis strategy

To test the specificity of a barrier or driver for the transition between two stages we made the following assumptions: A transition is supposed to have occurred when there is a high congruency between embracing two consecutive statements in the stage progression measure. For example: If a person both agrees strongly to *deciding what to do* and *how to do it*, it is assumed that this person is rather in the *how to do it* stage than the *deciding what to do* stage. If a person, however, embraces the first but not the second statement, he or she would more likely not have progressed to the latter stage. If this general logic is accepted, a barrier is a variable that reduces the link between two consecutive stage statements, whereas a driver is a variable that increases the strength of this link. In other words, important barriers and drivers are moderators of the relation between stage statements. For the analysis of stage specificity of the barriers and drivers, it was thus analyzed, which barriers and drivers moderated the stage-statement relations at which stage.

More technically, we considered a barrier or a driver relevant for progression through decision-making when the variable significantly moderated the strength of the relation between an earlier stage statement and a later stage statement (e.g., the relation between indicating that one is *in decision mode* and *deciding what to do*). To test this moderating effect, we regressed the later stage statement on the earlier stage statement, the barrier or driver and the interaction term between the two. The variables were mean-centered before the interaction term was calculated to avoid false multi-collinearity. Each barrier and each driver were tested in an individual analysis, resulting in 24 regression analyses per stage transition. For getting the estimates presented in the first line of Table 3 for example, the following regression analysis was conducted: The degree of agreeing that something needs to be done about the insulation standard was regressed on the agreement that there were no plans for upgrading insulation (reverse coded), the agreement to being unsure about the saving potential, and the interaction between the two, which is the mean-centered product term of the two variables. Barriers were expected to reduce the relation between the agreement to the earlier stage description (e.g., not being in decision mode) and the agreement to a later stage description (e.g., wanting to find out what to do). Drivers were expected to strengthen this relationship significantly. The results displayed in the tables are standardized regression coefficients, and we focus on the coefficients for the interaction terms. The significance levels are not adjusted for family-wise alpha error inflation (Bonferroni correction). Although repeating a statistical test increases the likelihood of false positive results and the p-levels are recommended to be adjusted accordingly, some authors argue against applying this procedure and rather take a substantial evaluation of the result pattern into account (Moran, 2003).

## Results

Table 2 displays the zero-order correlations between the four statements indicating the stages of decision-making. The reverse coded not being in the decision mode correlates with a medium effect size with deciding what to do. Deciding what to do correlates strongly with deciding how to do it; deciding how to do it correlates strongly with deciding how to implement.

**Table 2:** **Correlations between agreements to the statements indicating the different stages of decision-making (*N* = 3.787)**.

	**Not being in decision-mode (reverse coded)**	**Deciding what to do**	**Deciding how to do it**
**NOT BEING IN DECISION-MODE (REVERSE CODED)**			
Deciding what to do	0.425^***^		
Deciding how to do it	0.400^***^	0.805^***^	
Deciding how to implement	0.417^***^	0.722^***^	0.825^***^

*** p < 0.0010.

**Figure 2:**
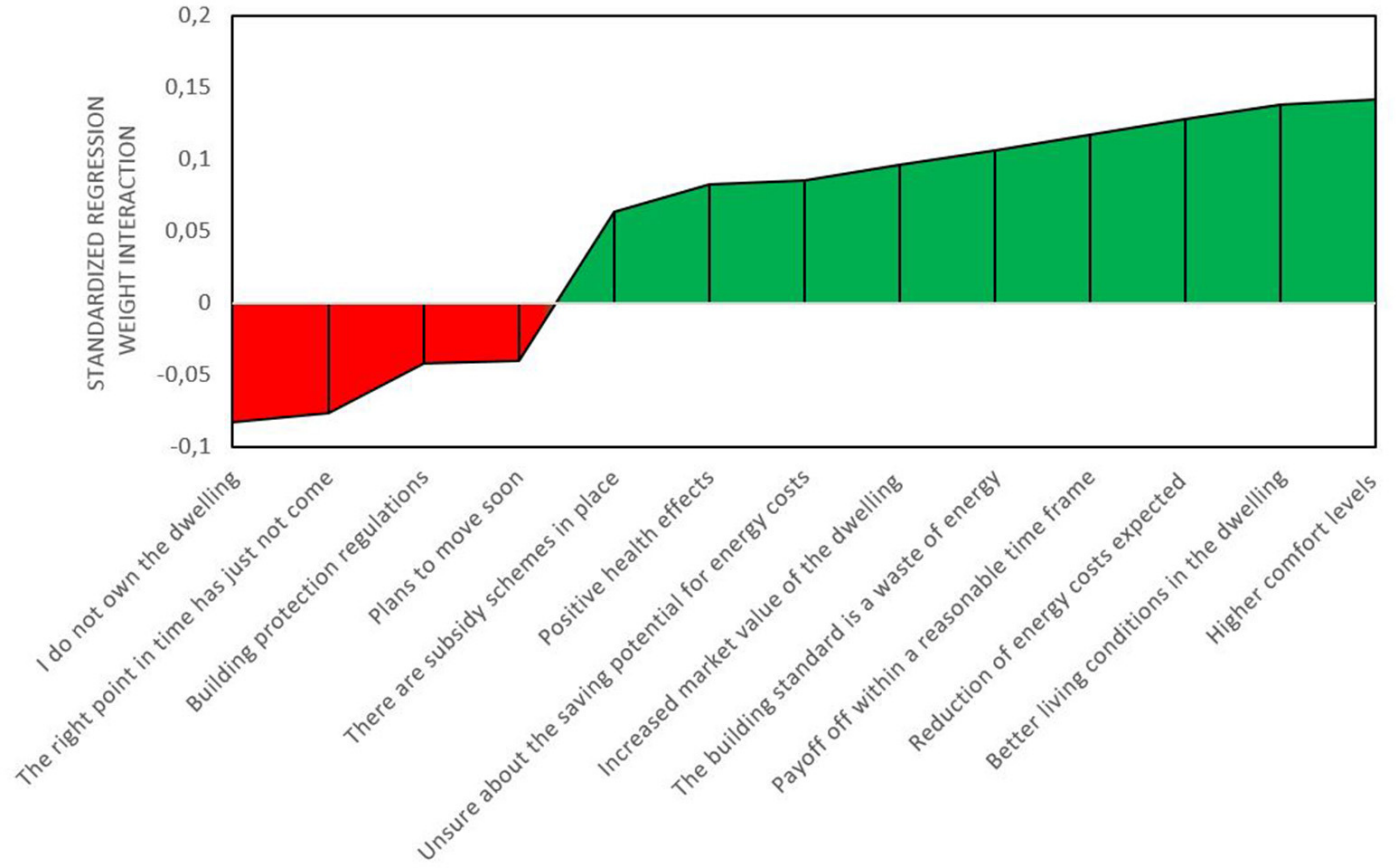
**Significant barriers and drivers for the transition from *not being in a decision mode* (reverse coded) to *deciding what to do***.

The first block of the moderation analyses (see Table 3) shows that link between not being in decision mode and deciding what to do is in general not affected by including the additional predictors (drivers and barriers). Four barriers have negative interaction terms (as expected), namely plans to move soon, not owning the dwelling, doubting that the right time has come yet, and building protection regulations. For all these barriers, the link between (reversed) *not being in the decision mode* and *deciding what to do* is weaker when the barrier is strong, indicating that it is less likely that a person has progressed to the latter stage. One aspect that was expected to be a barrier (being unsure about the saving potentials) had an unexpected positive interaction weight, indicating that high agreement to this statement goes along with stronger relations between the two stage statements. Almost all drivers had the expected positive interaction, which means making the relation between the reverse-coded “*not being in decision mode*” and “*deciding what to do*” stronger. The exceptions were easily accessible and trustworthy information. Figure 2 displays the significant interactions sorted by the size of their standardized regression weights.

**Table 3:** **Analysis of the moderating effects of barriers and drivers on the transition from *not being in decision mode* to *deciding what to do* (*N* = 3.787)**.

	**Not being in decision mode**	**Barrier/Driver**	**Interaction**
**BARRIERS**			
Unsure about the saving potential for energy costs after an upgrade	0.427^***^	0.124^***^	0.085^***^
Plans to move soon	0.422^***^	0.064^***^	−0.040^*^
I do not manage to make a decision for what to do	0.399^***^	0.196^***^	−0.006
I do not own the dwelling	0.417^***^	0.035^*^	−0.083^***^
The right point in time has just not come to upgrade	0.336^***^	−0.206^***^	−0.077^***^
Building protection regulations prevent me from upgrading	0.422^***^	0.090^***^	−0.042^*^
Not enough economic resources	0.418^***^	0.127^***^	0.038
Contractors which could do the job lack the necessary competencies	0.423^***^	0.098^***^	0.015
Depending on agreement with neighbors	0.426^***^	0.025	−0.008
Difficult to know if information about energy upgrades can be trusted	0.423^***^	0.059^***^	−0.013
Too much disturbance of the everyday life through such a project	0.427^***^	0.063^***^	0.000
Information about upgrading is difficult to find	0.419^***^	0.102^***^	0.011
Demands much time to supervise the contractors	0.426^***^	0.032^*^	−0.007
Negative experience from previous projects	0.424^***^	0.079^***^	−0.007
**DRIVERS**			
Reduction of energy costs expected after upgrade	0.349^***^	0.267^***^	0.128^***^
Increased market value of the dwelling expected after upgrade	0.352^***^	0.248^***^	0.096^***^
Payoff of the investment within a reasonable time frame	0.384^***^	0.182^***^	0.117^***^
Positive health effects expected after upgrade	0.391^***^	0.164^***^	0.082^***^
The building standard of the dwelling is perceived as a waste of energy	0.260^***^	0.356^***^	0.106^***^
Better living conditions in the dwelling expected after upgrade	0.308^***^	0.299^***^	0.138^***^
Higher comfort levels expected after upgrade	0.349^***^	0.235^***^	0.141^***^
Information about energy upgrade is easily accessible	0.426^***^	0.005	0.011
Information about energy upgrade is trustworthy	0.424^***^	0.011	0.033
There are subsidy schemes in place supporting the upgrade	0.427^***^	−0.013	0.063^**^

*** p < 0.001;

** p < 0.01;

* p < 0.05.

Displayed are standardized regression weights. Significance values are not Bonferroni-corrected.

**Figure 3:**
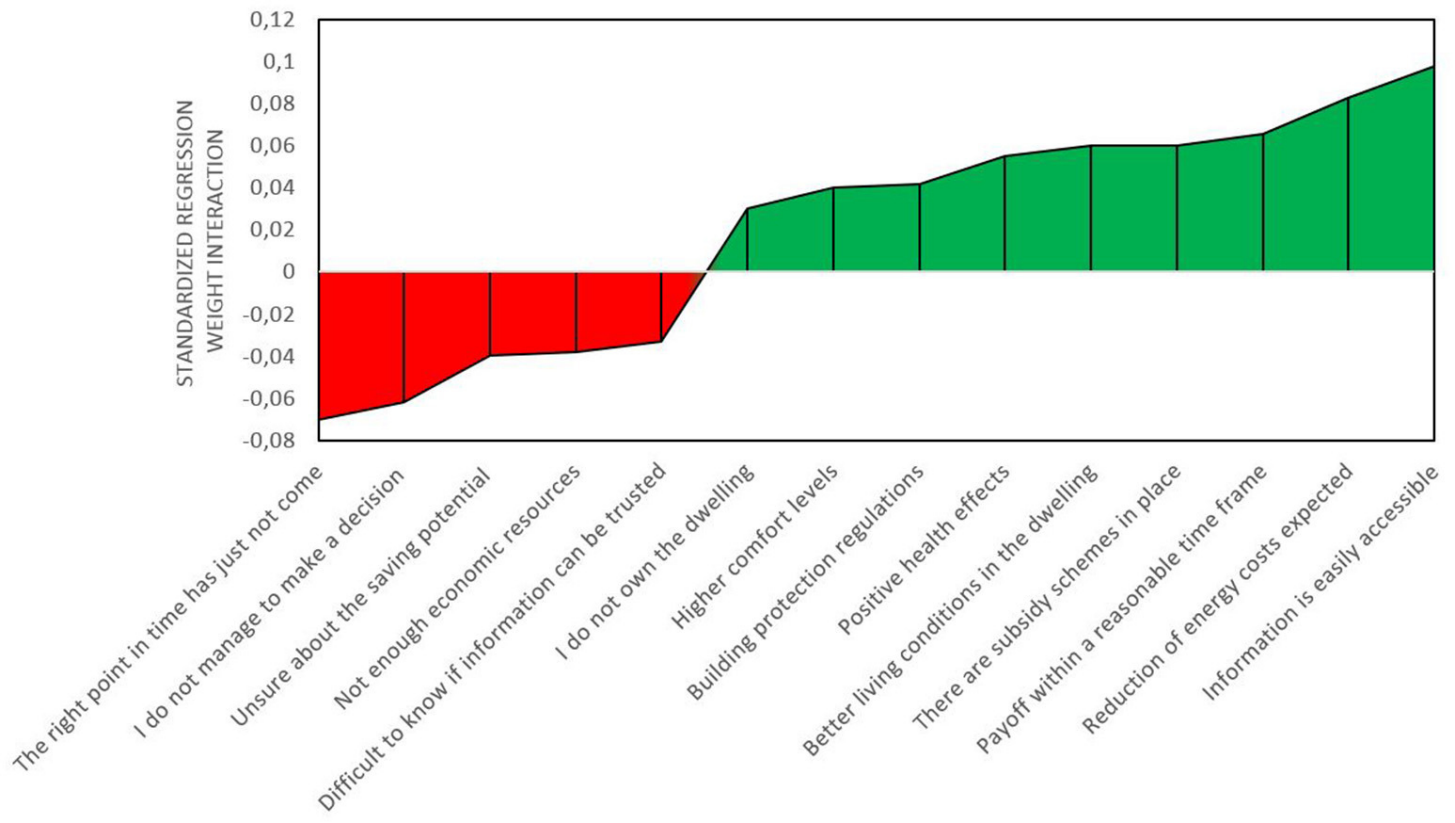
**Significant barriers and drivers for the transition from *deciding what to do* to *deciding how to do it***.

The second block of the moderation analyses (see Table 4) shows that also link between *deciding what to do* and *deciding how to do it* is in general not affected by including the additional predictors (drivers and barriers). Four barriers have negative interaction terms (as expected), namely not being able to make a decision, feeling that the right time has not come yet, not having enough economic resources, and not being sure how to validate the trustworthiness of information about energy upgrades. Two barriers (not owning the dwelling and building regulations) had an unexpected positive, but small interaction weights. Seven drivers had the expected positive interaction. The exceptions were expected increased market value, feeling the current insulation standard is a waste of energy, and trustworthy information. Figure 3 displays the significant interactions sorted by the size of their standardized regression weights.

**Table 4:** **Analysis of the moderating effects of barriers and drivers on the transition from *deciding what to do* to *deciding how to do it* (*N* = 3.787)**.

	**Deciding what to do**	**Barrier/Driver**	**Interaction**
**BARRIERS**			
Unsure about the saving potential for energy costs after an upgrade	0.815^***^	−0.043^***^	−0.040^*^
Plans to move soon	0.805^***^	0.002	0.010
I do not manage to make a decision for what to do	0.826^***^	−0.043^***^	−0.062^***^
I do not own the dwelling	0.805^***^	0.034^***^	0.030^*^
The right point in time has just not come to upgrade	0.747^***^	−0.097^***^	−0.070^***^
Building protection regulations prevent me from upgrading	0.800^***^	0.018^*^	0.042^*^
Not enough economic resources	0.813^***^	−0.028^**^	−0.038^*^
Contractors who could do the job lack the necessary competencies	0.804^***^	−0.002	0.014
Depending on agreement with neighbors	0.805^***^	−0.019^*^	0.003
Difficult to know if information about energy upgrades can be trusted	0.808^***^	−0.020	−0.033^*^
Too much disturbance of the everyday life through such a project	0.806^***^	−0.015	−0.000
Information about upgrading is difficult to find	0.808^***^	−0.009	−0.018
Demands much time to supervise the contractors	0.805^***^	−0.012	−0.005
Negative experience from previous projects	0.804^***^	0.005	0.017
**DRIVERS**			
Reduction of energy costs expected after upgrade	0.739^***^	0.100^***^	0.083^***^
Increased market value of the dwelling expected after upgrade	0.776^***^	0.056^***^	0.029
Payoff of the investment within a reasonable time frame	0.769^***^	0.068^***^	0.066^***^
Positive health effects expected after upgrade	0.777^***^	0.060^***^	0.055^***^
The building standard of the dwelling is perceived as a waste of energy	0.769^***^	0.063^***^	0.013
Better living conditions in the dwelling expected after upgrade	0.746^***^	0.078^***^	0.060^**^
Higher comfort levels expected after upgrade	0.774^***^	0.050^***^	0.040^*^
Information about energy upgrade is easily accessible	0.803^***^	0.063^***^	0.098^***^
Information about energy upgrade is trustworthy	0.803^***^	0.013	0.028
There are subsidy schemes in place supporting the upgrade	0.801^***^	0.010	0.060^**^

*** p < 0.001;

** p < 0.01;

* p < 0.05.

Displayed are standardized regression weights. Significance values are not Bonferroni-corrected.

**Figure 4:**
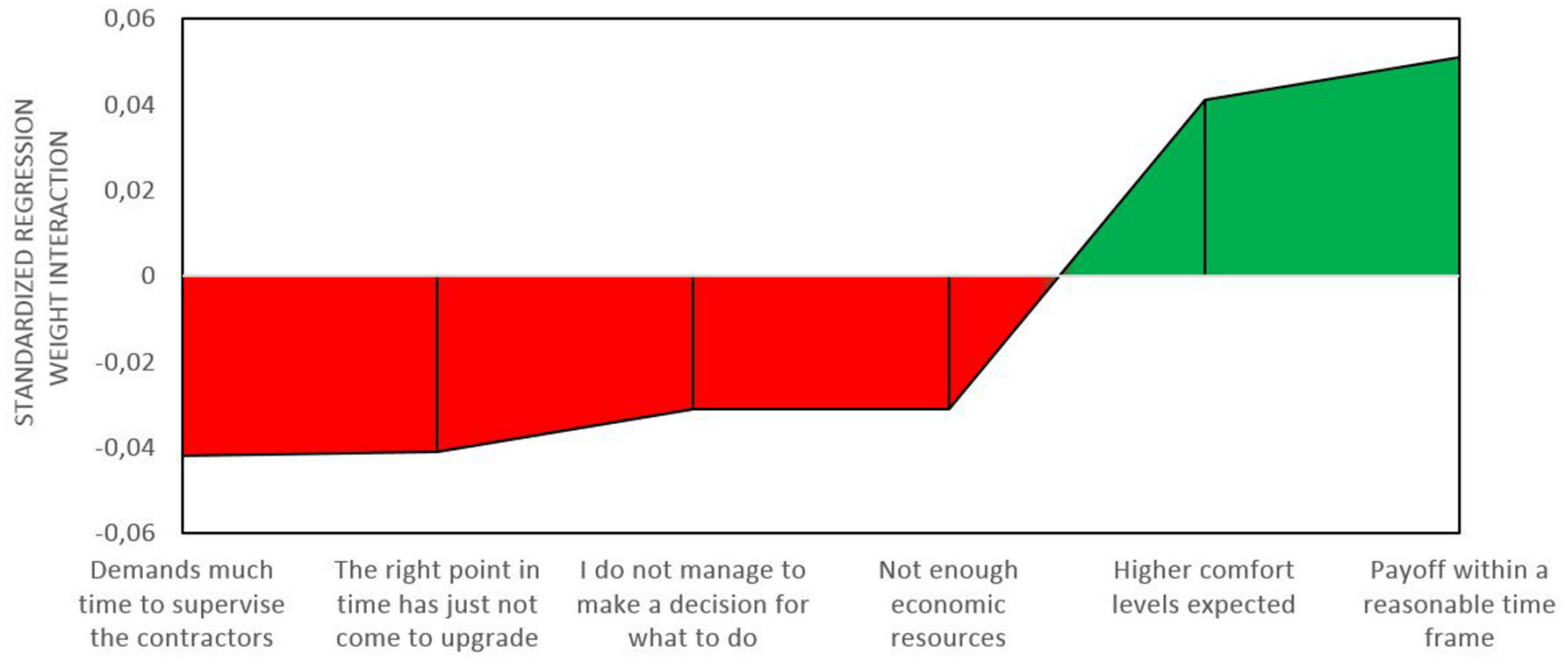
**Significant barriers and drivers for the transition from *deciding how to do it* to *planning how to implement***.

The last block of the moderation analyses (see Table 5) shows that also link between *deciding how to do it* and *planning how to implement* is not affected by including the additional predictors (drivers and barriers). Four barriers have negative interaction terms, namely not being able to make a decision, feeling that the right time has not come yet, not having enough economic resources, and anticipating high demands to supervise the contractors. Two drivers had the expected positive interaction, namely expecting a payoff of the investment within a reasonable time frame and expected higher comfort levels. Figure 4 displays the significant interactions sorted by the size of their standardized regression weights.

**Table 5:** **Analysis of the moderating effects of barriers and drivers on the transition from *deciding how to do it* to *planning implementation* (*N* = 3.787)**.

	**Deciding how to do it**	**Barrier/Driver**	**Interaction**
**BARRIERS**			
Unsure about the saving potential for energy costs after an upgrade	0.826^***^	−0.011	−0.024
Plans to move soon	0.826^***^	−0.010	−0.000
I do not manage to make a decision for what to do	0.827^***^	−0.005	−0.031^*^
I do not own the dwelling	0.825^***^	−0.011	−0.031
The right point in time has just not come to upgrade	0.775^***^	−0.087^***^	−0.041^*^
Building protection regulations prevent me from upgrading	0.825^***^	−0.003	0.008
Not enough economic resources	0.827^***^	−0.022^*^	−0.031^*^
Contractors who could do the job lack the necessary competencies	0.823^***^	0.014	0.021
Depending on agreement with neighbors	0.826^***^	−0.011	0.015
Difficult to know if information about energy upgrades can be trusted	0.825^***^	−0.002	−0.012
Too much disturbance of the everyday life through such a project	0.825^***^	−0.009	−0.013
Information about upgrading is difficult to find	0.825^***^	0.008	−0.009
Demands much time to supervise the contractors	0.824^***^	−0.032^**^	−0.042^**^
Negative experience from previous projects	0.826^***^	−0.004	0.010
**DRIVERS**			
Reduction of energy costs expected after upgrade	0.807^***^	0.054^***^	0.001
Increased market value of the dwelling expected after upgrade	0.796^***^	0.064^***^	0.023
Payoff of the investment within a reasonable time frame	0.790^***^	0.073^***^	0.051^**^
Positive health effects expected after upgrade	0.804^***^	0.057^***^	0.026
The building standard of the dwelling is perceived as a waste of energy	0.793^***^	0.070^***^	0.004
Better living conditions in the dwelling expected after upgrade	0.778^***^	0.078^***^	0.036
Higher comfort levels expected after upgrade	0.787^***^	0.073^***^	0.041^*^
Information about energy upgrade is easily accessible	0.823^***^	0.004	0.013
Information about energy upgrade is trustworthy	0.827^***^	−0.003	−0.023
There are subsidy schemes in place supporting the upgrade	0.825^***^	0.016	0.020

*** p < 0.001;

** p < 0.01;

* p < 0.05.

Displayed are standardized regression weights. Significance values are not Bonferroni-corrected.

**Figure 5:**
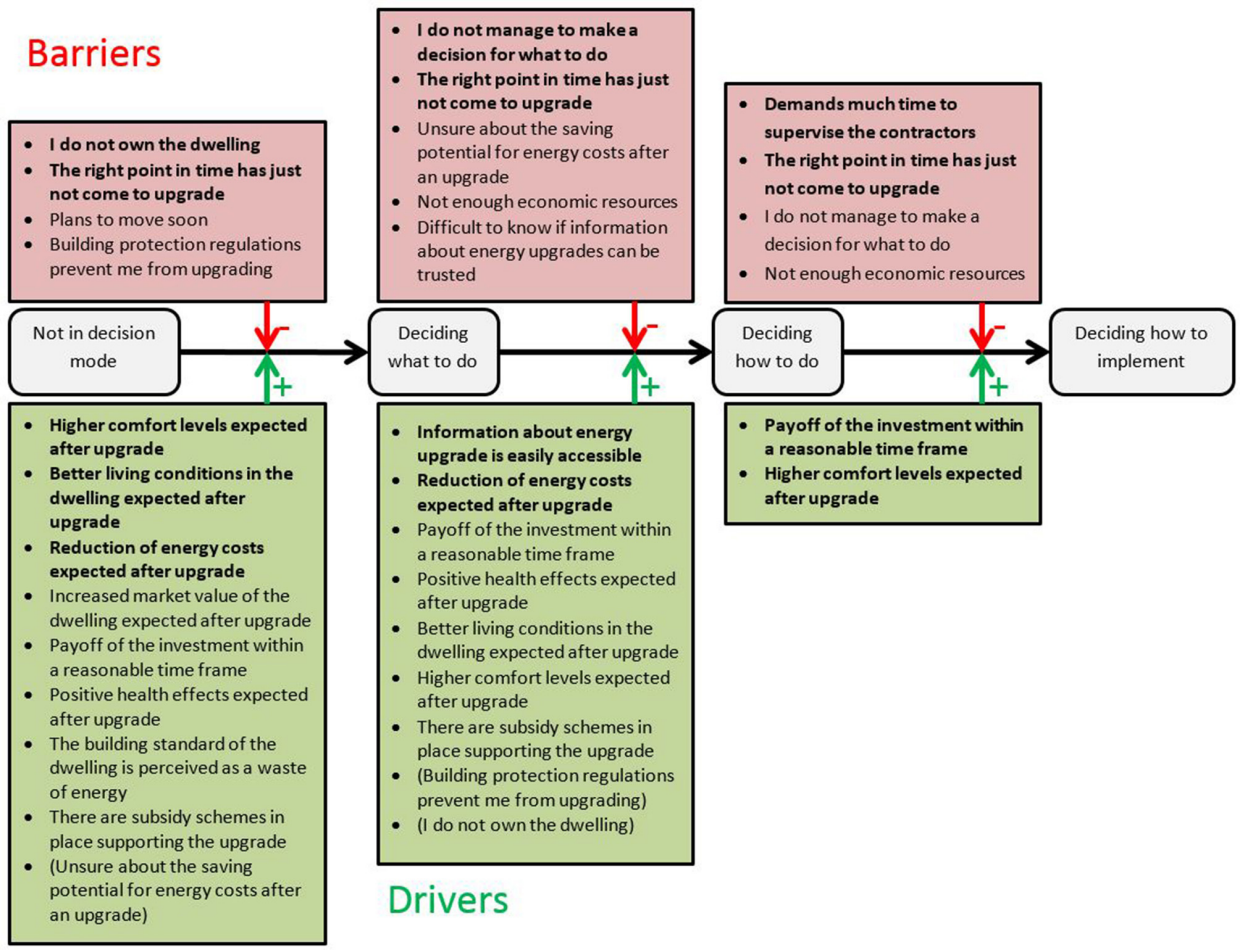
**Overview of the structure of barriers and drivers**.

Figure 5 summarizes the findings from the analyses by displaying all significant drivers and barriers with the most important in each transition printed in bold.

## Discussion

The study shows that a stage-specific analysis of barriers and drivers of decision-making toward energy efficiency upgrades gives insights that would be lost if barriers and drivers were analyzed without taking into account how far a person has come in decision-making. In doing this, the study goes beyond other studies of barriers and drivers conducted in other countries (Jakob, 2007; Nair et al., 2010; Zundel and Stieß, 2011) which studies similar sets of barriers and drivers. The findings support the value that stage-based models might have for designing marketing or intervention strategies (Bamberg, 2007, 2012) in this domain. When comparing the results of the analyses with the hypotheses in Table 1, most expectations have been confirmed. For the transition from *not being in decision mode* to *deciding what to do*, all barriers we expected to be relevant showed to be relevant except negative experience from previous projects. Also for the drivers, our hypotheses were confirmed. However, we did not expect that existing subsidy schemes can also be a trigger for starting to think about energy upgrades, which might be explained by that a media connected to such subsidies might create attention to the topic. In the second transition from *deciding what to do* to *deciding how to do it*, we expected coordination with neighbors and difficulties to find information to be relevant barriers, which did not show in our results. Otherwise, our expectations were confirmed. The expected facilitating influence of trustworthy information was also not found, but all other hypotheses about relevant drivers for the second transition were confirmed. In the last transition from *deciding how to do it* to *deciding how to implement*, the pattern confirms our expectations for the drivers. For barriers, however, our hypotheses were not confirmed. Whereas we expected the lack of competence of contractors, the need for an agreement with neighbors and too much disturbance to be relevant, this was not supported by the data. On the other hand, we did not expect that decision-making difficulties and a feeling of the right point in time is yet to come being relevant that late in the process after so many previous decisions. Probably, the inability to make a decision is located on different levels of specificity in different stages. Whereas some people are not able to make even a general decision if to do something, whereas others can do that but are stopped later in the process when deciding how to implement their plans. A lack of economic resources as a barrier at such a late stage was also surprising for us and seems to indicate that economic evaluations of alternatives happen at a later stage when the alternatives become concrete and get a “price tag.”

It is not particularly surprising, that people who do not own the dwelling they live in do not even consider larger investments in energy upgrades. The same is true for people who plan to move soon or people living in protected buildings in which strict regulations are a structural barrier. Later in the decision-making process, other barriers become more important though: Not being able to make a decision is the main barrier for deciding which energy efficiency measure(s) to go for in the refurbishment project. Also, insecurity about the information related to the energy efficiency measures is relevant at this stage. Even further along in the process, anticipating implementation problems like having to supervise the contractors become most relevant. Addressing the barriers stage-specific makes, therefore, a lot of sense. A person not yet engaged in the decision-making most likely not be interested in information, how to select reliable contractors—he or she might even get irritated by this information realizing that contractors actually might become a problem. Interestingly, lack of economic resources is not a barrier at the beginning of the process, but it gets the more relevant the closer to implementation of the plans the decision-making process is, indicating that early in the process, focus in campaigns should rather be put on other aspects. A barrier that appears at every stage of decision-making is the feeling “that the right time for the energy efficiency upgrade has not come yet.” The need for new ways of making people realize when this point in time has come is a clear recommendation from the survey. The right time for energy upgrades in the Norwegian context at least is when people plan a major refurbishment, so campaigns need to focus on targeting people in such a situation, for example through providers of refurbishment services, tools or material. A “right point in time” is also when emergency refurbishment measures have to be implemented (when for example the façade is leaking and needs to be replaced). In this case, it relies strongly on the contractor recommending the energy upgrades and making funding resources quickly available.

On the level of drivers, the picture is even more diverse. Relevant drivers are health, comfort, and economy (payoff, lower energy costs, higher market value, and subsidy) related. Some are even on the moral side, namely a feeling that the energy use of the building is a waste, apparently appealing to people with strong frugality motivations (Fujii, 2006). Overall, drivers that related to comfort and health appear to be more important than economic drivers. They are relevant at all stages of decision-making and should thus be addressed at all levels: first on the general level (“improving the energy efficiency of your house increases your well-being and comfort”), then at the level of measures and implementation (“increasing the insulation level of your walls increases your comfort”). It is interesting that the economic payoff grows in importance during the decision-making process, whereas existing subsidy schemes are more important in the earlier than in later stages, which might indicate that a subsidy can motivate during deciding if and in what kind of efficiency upgrade to invest whereas later a more thorough calculation of the payoff happens.

The unexpected positive signs for some of the barriers are also interesting to look at in more detail. That “being unsure about the saving potential” behaves rather like a driver than a barrier might indicate that people that are unsure about saving potentials do assume a saving potential, but are not sure at this early stage, how big it might be. The two positive signs for “not owning the dwelling” and “building protection regulations” between *deciding what to do* and *deciding how to do it* are harder to explain. Maybe, people who did hop over the first threshold and entered decision-making in spite of not owning or being affected by strict building protection regulations are more motivated than usual people to proceed.

The findings of this study have implications for intervention programs. Knowing about stage specificity of certain barriers and drivers allows for designing interventions specific to the needs at certain points of the decision-making process. People in the early stage of decision making can be addressed by triggering their desire for higher comfort and better living conditions, whereas people not owning the dwelling or planning to move can be excluded from marketing campaigns for energy efficiency upgrades. Later in the process, accessible information, subsidy schemes, and cost related information are good motivators to proceed. Tools for enhancing decision-making could also be useful, to reduce procrastination tendencies. In the last stage of implementing, the perception of high demands for supervision is crucial to address.

Despite its useful and promising results the study has limitations which should be addressed in future studies. First, the study is based on cross-sectional data, which regarding a dynamic model describing progression through stages limits the insights that could be derived. This has also been outlined as a general weakness of studies in this domain (Wilson et al., 2013). Especially the non-linearity of the process, which was well described in the longitudinal study by Klöckner (2014a), is not sufficiently represented in the results of the present study. The process of decision-making about such a big investment is hardly linear, loops and relapses are likely as Klöckner (2014a) demonstrated. A future study should follow people longitudinally through such a process to capture such non-linear dynamics and especially the mechanisms of overcoming barriers. Second, like all other studies known to us in this domain, also our study did ignore the within-household dynamics of decision-making. It would be interesting in future studies to not only capture the dynamics over time but also within the household, which usually includes at least two decision-makers. Third, the study did not record the last step of the decision process, namely if the decided measures were actually implemented at some point. This also reduces the generalizability of the results, since the gap between self-reported intentions and actual behavior is well known. Future studies should address this limitation by following the respondents for several years to see if decisions are implemented and how strong the relation between implementation intentions and behavior is. Fourth, the correlations between the statements regarding stage 2–4 are high, which indicates that the stages are probably not distinct enough or the measurement instrument did not manage well enough to separate them from each other. Future research should focus on exploring the distinctness of the stages proposed in this study further, especially the “*deciding how to do it*” stage which sits between the “*deciding what to do*” and “*deciding how to implement*” might have blurred the borders between the stages. Finally, the results are limited to the Norwegian context, although it can be expected that many of the found barriers and drivers can be generalized to other (at least western) countries. Similar studies from Sweden, Switzerland and Germany, show many of the same barriers and drivers to be relevant (Jakob, 2007; Nair et al., 2010; Zundel and Stieß, 2011). However, replication in other countries and cultural contexts would strengthen the findings, especially since the other studies did not focus on stages of decision-making.

## Author contributions

Both authors of the paper were involved in conception and design of the study, analyzing the data, and writing up the results into a paper. Both authors agree to be accountable of all aspects of the work.

## Funding

This research was funded by the Norwegian Energy Agency Enova.

### Conflict of interest statement

The authors declare that the research was conducted in the absence of any commercial or financial relationships that could be construed as a potential conflict of interest.
